# The Diarylheptanoid Hirsutenone Sensitizes Chemoresistant Ovarian Cancer Cells to Cisplatin via Modulation of Apoptosis-inducing Factor and X-linked Inhibitor of Apoptosis*

**DOI:** 10.1074/jbc.M113.513879

**Published:** 2013-11-18

**Authors:** Lee Farrand, Ji Young Kim, Sanguine Byun, Akechai Im-aram, Jihoon Lee, Jeong-Yong Suh, Ki-Won Lee, Hyong Joo Lee, Benjamin K. Tsang

**Affiliations:** From the ‡ World Class University Major in Biomodulation, Department of Agricultural Biotechnology, College of Agriculture and Life Sciences, Seoul National University, Republic of Korea,; the Departments of § Obstetrics and Gynaecology and; ‖ Cellular and Molecular Medicine, University of Ottawa, Ottawa, Ontario K1N 6N5, Canada, and; the ¶ Chronic Disease Program, Ottawa Hospital Research Institute, Ottawa, Ontario K1N 6N5, Canada

**Keywords:** Akt, Chemoresistance, Ovarian Cancer, p53, XIAP, AIF, Hirsutenone

## Abstract

Cisplatin (CDDP) and its derivatives are considered first-line treatments for ovarian cancer (OVCA). However, despite initial results that often appear promising, in most cases patients will return with recurrent disease that fails to respond to further chemotherapy. We assayed a number of food phytochemicals with reported PI3K inhibitory ability to identify candidates that can influence CDDP treatment outcomes in chemoresistant OVCA cell lines. A direct comparison revealed that the diarylheptanoid hirsutenone from the tree bark of *Alnus hirsuta* var. sibirica was superior at inducing CDDP sensitivity in a number of chemoresistant cancer cell lines. Whereas hirsutenone treatment activated p53, its modest efficacy in *p53*-mutant and -null cell lines suggested the existence of a p53-independent mode of action. Further investigation revealed that hirsutenone causes CDDP-dependent apoptosis in chemoresistant cells by ubiquitin-proteasome-dependent X-linked inhibitor of apoptosis degradation and by enhancing the translocation of apoptosis-inducing factor from the mitochondria to the nucleus. This was found to be, at least in part, under the influence of upstream Akt activity, linking hirsutenone-dependent PI3K inhibition with downstream effects on apoptosis-inducing factor, X-linked inhibitor of apoptosis, and apoptosis. Our findings provide rationale for further investigation of the effects of hirsutenone on chemoresistant OVCA in clinical studies.

## Teaser

**Background:** Resistance of ovarian cancer cells to chemotherapy is a major therapeutic problem.

**Results:** Hirsutenone induces cisplatin sensitivity via p53, X-linked inhibitor of apoptosis protein, and apoptosis-inducing factor.

**Conclusion:** Hirsutenone sensitizes resistant ovarian cancer cells to cisplatin.

**Significance:** Co-treatment with hirsutenone may have the potential to overcome chemoresistance.

## Introduction

Ovarian cancer (OVCA)^2^ is the fifth leading cause of cancer-related deaths in women worldwide (1). Cisplatin (CDDP: *cis*-diamminedichloroplatinum) and its derivatives (including carboplatin and oxaliplatin) are considered first-line treatments for OVCA and function by inducing apoptosis and triggering cell cycle arrest (2). In the majority of cases, however, recurrent disease emerges after initial treatment rounds and fails to respond to further chemotherapy even at higher dosages. This phenomenon, known as chemoresistance, presents a significant medical problem for the treatment of cancer types that are frequently diagnosed late, including cancers of the ovary, pancreas, and bowel.

Chemoresistance enables cancer cells to evade apoptotic stimuli and arises from the dysregulation of signaling factors responsible for inducing cell death (3). p53 is a prominent tumor suppressor with transcription-dependent and -independent modes of action that lead to the activation of apoptosis (4, 5). However, by conservative estimates, at least half of all cancers of the ovary are defective for *p53*, implying that the gene is either mutated or null (6). Therefore, a significant requirement exists for therapeutic strategies that influence p53-independent pathways of apoptotic induction as well as to address the problem of p53 deficiency and lack of responsiveness.

A major caspase-independent mechanism of cell death is regulated by aoptosis-inducing factor (AIF), a flavoprotein normally localized to the outer mitochondrial membrane (7). Upon release from the mitochondria, AIF translocates to the nucleus where it induces DNA fragmentation and chromatin condensation. AIF is negatively regulated by the X-linked inhibitor of apoptosis protein (XIAP) which is present in the cytosol and nucleus (8). XIAP is frequently maintained at high levels in chemoresistant OVCA cells and acts by suppressing both caspase activity and AIF via polyubiquitination (9, 10). The suppression of XIAP therefore represents a potential strategy for addressing chemoresistance (11). XIAP has been shown to be regulated upstream by the PI3K/Akt pathway (12), and numerous synthetic PI3K inhibitors, including LY294002, have been developed in the hopes of blocking this pathway and improving treatment outcomes. However, the vast majority of synthetic PI3K inhibitors have proven too cytotoxic for general use, likely due to their broad specificity toward undesired cellular targets (13).

Food phytochemicals are bioactive molecules that can be extracted from natural plant-based food sources. Recent research has revealed that some candidates exhibit remarkable potency at inhibiting pathways relevant to cancer prevention and chemoresistance (14–16). Hirsutenone is a diarylheptanoid found commonly in the bark of *Alnus hirsuta* var. sibirica (17). Evidence suggests that hirsutenone exhibits numerous bioactive properties, including the ability to suppress T cell activation and induce TRAIL-dependent apoptosis (18, 19). However, the effect of hirsutenone on CDDP sensitivity in cancer cells has not previously been investigated.

The objective of the present study was to investigate the effects of hirsutenone treatment alone and in combination with CDDP on chemoresistant OVCA cells and its mechanisms of action. We hypothesize that hirsutenone induces CDDP sensitivity, in part, via down-regulation of Akt function, leading to the degradation of XIAP and AIF-dependent apoptosis. Our findings support the contention that hirsutenone could be useful in the treatment of chemoresistant OVCA.

## EXPERIMENTAL PROCEDURES

### 

#### 

##### Reagents

CDDP, DMSO, Hoechst 33258, lactacystin, apigenin, luteolin, myricetin, piceatannol, quercetin, and epoxomicin were purchased from Sigma-Aldrich. Cyanidin and delphinidin were purchased from ChromaDex (Irvine, CA). 7,3,4-Trihydroxyisoflavone and 6,7,4-trihydroxyisoflavone were purchased from Indofine Chemical Company (Hillsborough, NJ). Hirsutenone was purchased from Chemfaces (Daejeon, Republic of Korea). Purified recombinant human whole PI3K protein was purchased from Millipore. Mouse monoclonal p53 (DO-1), MDM2, AIF, and TOM20 antibodies were from Santa Cruz Biotechnology. GAPDH, rabbit monoclonal anti-phospho-Ser^15^-p53, Akt, phospho-Ser^473^-Akt, phospho-Thr^308^-Akt, and HA antibodies, as well as AIF and scrambled siRNA constructs were from Cell Signaling Technology. Anti-XIAP and -caspase-3 antibodies were from Abcam. Peroxidase-conjugated goat anti-mouse and goat anti-rabbit immunoglobulin were purchased from Bio-Rad. Alexa Fluor 488 and 594 secondary antibodies, Lipofectamine 2000 transfection reagent, RNase A, TEMED, RPMI 1640 medium, and DMEM/F12 culture medium, and fetal bovine serum were from Invitrogen. Complete Mini Protease inhibitor mixture tablets and PhosStop phosphatase inhibitor mixture tablets were obtained from Roche Applied Sciences. Pc-CTL, Pc-Myr-Akt, and Pc-XIAP recombinant plasmids and the adenoviral (wt-p53, GFP) constructs were synthesized by Vector Biolabs. The MTS assay kit was purchased from Promega.

##### Cell Lines and Culture

CDDP-sensitive (OV2008 (wt-p53), A2780s (wt-p53), OVCAR-432 (*p53*-mutant)) and -resistant (C13*, Hey, OVCAR-433 (wt-p53), A2780cp, Occ-1 (*p53*-mutant), and SKOV3 (*p53*-null)) human OVCA cell lines were gifts from Drs. Rakesh Goel and Barbara Vanderhyden (Ottawa Hospital Cancer Center, Ottawa, ON, Canada) and cultured previously as reported (19, 20). OV2008 and C13 cells were cultured in RPMI, whereas OVCAR-433, A2780s, A2780cp, Hey, Occ-1, SKOV3, and OVCAR-432 were cultured in DMEM, supplemented with 10% FBS.

##### In Vitro PI3K Assay

*In vitro* PI3 kinase assays were carried out as described previously (21). Active PI3K protein (100 ng) was incubated (10 min, 30 °C) with the indicated test compounds. The mixture was then incubated (5 min, room temperature) with phosphatidylinositol (20 μl, 0.5 mg/ml, Avanti Polar Lipids, Alabaster, AC) for an additional 10 min at 30 °C in reaction buffer (100 mm Hepes (pH 7.6), 50 mm MgCl_2_, 250 μm ATP containing 10 μCi of [γ-^32^P]ATP). The reaction was stopped by the addition of HCl (4 n, 15 μl) and chloroform:methanol (1:1, 130 μl). After vortexing for 5–10 s, 30 μl of the chloroform phase was spotted onto a 1% potassium oxalate-coated silica gel chromatography plate, and the resulting ^32^P-labeled phosphatidylinositol-3,4,5-trisphosphate was separated and visualized by autoradiography.

##### MTS Assay and Hoechst 33258 Staining

Cell viability was assessed with the MTS assay (22). Cells were seeded for 15 h in 96-well plates in RPMI 1640 medium or DMEM with 10% FBS before replacement with respective serum-free media containing the indicated food phytochemicals (10 μm) in the presence or absence of CDDP (10 μm, 24 h), after which tetrazolium compound (Promega) was added to the cultures for an additional 2 to 4 h, according to the manufacturer's instructions. Absorbance at 490 nm was determined by a Bio-Rad X-Mark Microplate Analyzer. Apoptosis was assessed morphologically, as described previously (20). Briefly, cells were removed by trypsinization (0.05% trypsin, 0.53 mm EDTA; 37 °C, 1 min) at the end of the culture period, and then the trypsin was neutralized with RPMI 1640 medium containing 10% FBS, before washing in ice-cold PBS. Cells were fixed in neutral-buffered 10% formalin and stained at 4 °C overnight with the nuclear dye Hoechst 33258 (6.25 ng/ml). Cells were spotted onto slides and assessed for typical apoptotic nuclear morphology (nuclear shrinkage, condensation, and fragmentation) under a fluorescence microscope fitted with a DAPI filter. At least 400 cells were counted for each treatment group, and the process was blinded to avoid experiment bias (11).

##### Adenoviral Infection

Cells were infected with adenoviral constructs containing wt-p53 (multiplicity of infection = 10, 6 h) or GFP (as control) with an infection efficiency of >80%, as described previously (23), and successful forced expression was confirmed by Western blotting.

##### Immunoblotting, Immunoprecipitation, and Ubiquitination Assays

Immunoblotting was performed as described previously (24). All primary antibodies were used at 1:1000 dilution, except GAPDH (1:10,000) and p53 (DO-1; 1:5000). Band densities were analyzed and quantified using a Bio-Rad ChemiDoc XRS+ and Image Lab V3.0. For immunoprecipitation, 2.2 ×10^6^ cells were rinsed in 250 μl of lysis buffer and centrifuged, before supernatant (200 μl) was incubated with protein A Dynabeads (Invitrogen) coated with antibodies specific for the target proteins (2 μg/200 μl; 1 h, room temperature) and immunoprecipitated overnight (4 °C). The beads were pelleted (9000 × *g* 10 min), resuspended in Laemmli sample buffer (2×; 40 μl; Bio-Rad), boiled (10 min), and loaded onto 9% SDS-PAGE, according to the manufacturer's instructions. For the XIAP ubiquitination assay, OV2008 cells were transfected with HA-ubiquitin plasmids (1 μg) (Addgene) for 48 h using Lipofectamine 2000. Following treatment with hirsutenone (10 μg) for the indicated time points, cells lysates were analyzed by immunoblotting.

##### Immunofluorescence Microscopy

At the end of the culture period, cells on 8-well chamber slides (BD Biosciences) were fixed with 4% paraformaldehyde, washed with PBS, and incubated with Triton X-100 (0.2%, 10 min) before incubation with the indicated primary antibodies (1:100 dilution for AIF, 1:250 dilution for TOM20) in Dako Antibody Diluent, Invitrogen). Cells were then incubated with fluorescence-conjugated secondary antibodies (1:500 in Dako Antibody Diluent, room temperature; Invitrogen Alexa Fluor 488; catalog A11008 for TOM20, or Alexa Fluor 594; catalog A11032 for AIF) and stained with ProLong Gold Antifade Reagent (Invitrogen) with DAPI (blue, nuclear stain). Coverslips were fixed, and cells were imaged immediately with a Zeiss LSM700 confocal scanning microscope equipped with a Zeiss T-PMT digital camera. Negative control (nonspecific mouse and rabbit IgGs) were used to confirm the reliability of signal localization. The use of confocal microscopy allowed us to distinguish co-localized nuclear DAPI and AIF signal, as the laser cross-section taken through the *z* axis of the cell allowed image splices to be taken within the nucleus (indicating that AIF was indeed within the nucleus as opposed to being present on the surface of the nuclear membrane).

##### Statistical Analysis

Results are expressed as the mean ± S.E. of at least three independent experiments. Statistical analysis was carried out by one-way, two-way, or three-way analysis of variance, using SigmaPlot software (Versions 12; Systat Software, Chicago, IL). Differences between multiple experimental groups were determined by the Bonferroni post hoc test. Statistical significance was inferred at *p* < 0.05.

## RESULTS

### 

#### 

##### Piceatannol, Hirsutenone, Delphinidin, and Cyanidin Are Potent ATP-competitive Inhibitors of PI3K Activity in Vitro

**FIGURE 1.**
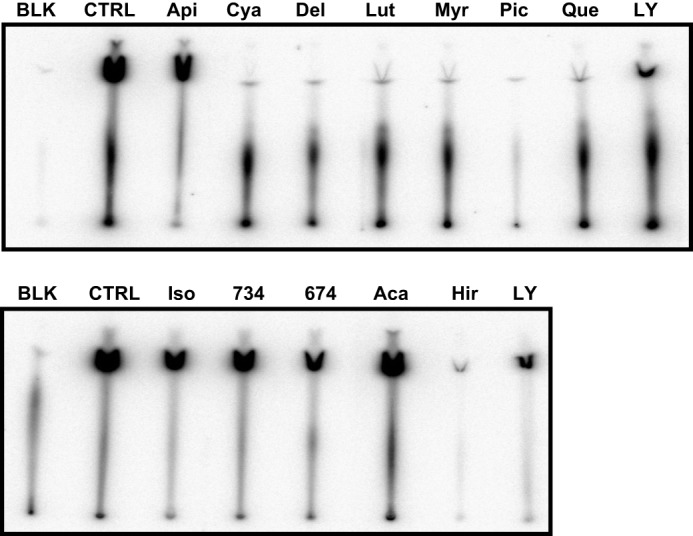
***In vitro* kinase assay data showing inhibitory properties of various food phytochemicals with reported PI3K inhibitory property.** PI3K protein was preincubated with the indicated test compounds for 10 min at 30 °C and then incubated with phosphatidylinositol substrate and 10 μCi of [γ-^32^P]ATP for an additional 10 min at 30 °C. The resulting ^32^P-labeled phosphatidylinositol 3-phosphate was measured as described under “Experimental Procedures.” LY294002 was used as a positive control. *BLK*, blank without PI3K, includes substrate and ATP; *CTL*, control, PI3K without sample; *Api*, apigenin; *Cya*, cyanidin; *Del*, delphinidin; *Lut*, luteolin; *Myr*, myricetin; *Pic*, piceatannol; *Que*, quercetin; *LY*, LY294002; *Iso*, isorhamnetin; *734*, 7,3,4′,4′-trihydroxyisoflavone; *674*, 6,7,4′,4′-trihydroxyisoflavone; *Aca*, acacetin; *Hir*, hirsutenone.

Using a library of phytochemicals with reported PI3K inhibitory activity, we first directly compared the relative ability of these compounds (at 10 μm) with inhibit kinase activity, using human recombinant PI3K (Millipore) in buffer (*ex vivo*). Piceatannol, hirsutenone, delphinidin, and cyanidin exhibited the strongest inhibition of PI3K activity and were more effective than the synthetic PI3K inhibitor LY294002, used as a positive control at the same concentration (Fig. 1). Based on these *in vitro* findings, we selected these four compounds for further study.

##### Hirsutenone Enhances the Effects of CDDP Treatment in Chemoresistant OVCA

**FIGURE 2.**
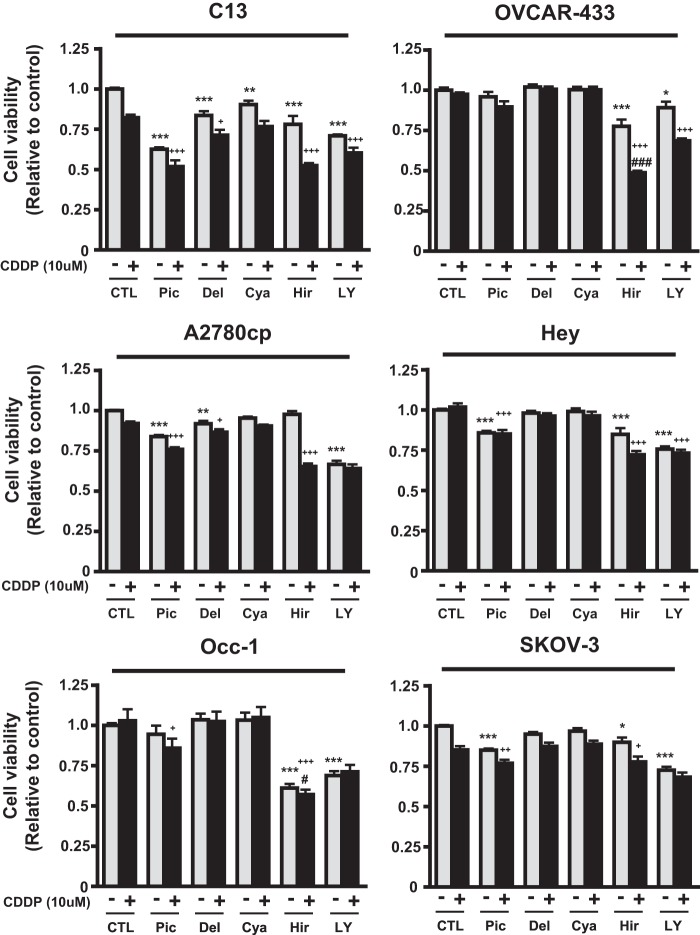
**Comparison of the influence of food phytochemicals alone or in combination with CDDP on OVCA cell viability *in vitro*.** Compounds with reported PI3K-inhibitory ability (10 μm) were assessed in the presence or absence of CDDP (10 μm; 24 h). Hirsutenone was more potent in sensitizing chemoresistant p53 wild-type OVCA (OVCAR-433, C13, and Hey) to CDDP. Hirsutenone decreased cell viability in *p53*-null/*p53-*mutant chemoresistant OVCA. DMSO was used as vehicle control; 24 h; *, *p* < 0.05; **, *p* < 0.01; ***, *p* < 0.001 (*versus* respective DMSO control). +, *p* < 0.05; ++, *p* < 0.01; +++, *p* < 0.001 (*versus* respective CDDP-only treatment). ###, *p* < 0.05 (*versus* respective LY294002 plus CDDP treatment). Results are expressed as mean ± S.E. (*error bars*; *n* = 3 independent experiments).

We used MTS assays to compare the effects of the food phytochemicals piceatannol, delphinidin, cyanidin, and hirsutenone (10 μm) alone and in combination with CDDP (10 μm) on the viability of a number of well established OVCA cell lines *in vitro*. The synthetic compound LY294002 (10 μm) was used as a positive control. Compared with piceatannol, hirsutenone was less effective in enhancing the cytotoxic effects of CDDP in chemosensitive cells (OV2008, A2780s, and OVCAR-432). In contrast, hirsutenone was the most effective compound overall for sensitizing chemoresistant OVCA cells irrespective of their *p53* status (wild-type *p53* (C13*, OVCAR-433; Fig. 2); mutant *p53* (A2780cp, Hey, Occ-1), and *p53*-null (SKOV3) (Fig. 2)), suggesting that the action of hirsutenone may occur by both p53-dependent and -independent means. In all cases for chemoresistant OVCA cells, hirsutenone was either equal to or more potent than the tested alternatives in enhancing the cytotoxic effects of CDDP.

##### Hirsutenone Induces Chemosensitivity in Chemoresistant OVCA Cells to a Greater Extent in p53 Wild-type Cells Than in Null/Mutant p53 Cells

**FIGURE 3.**
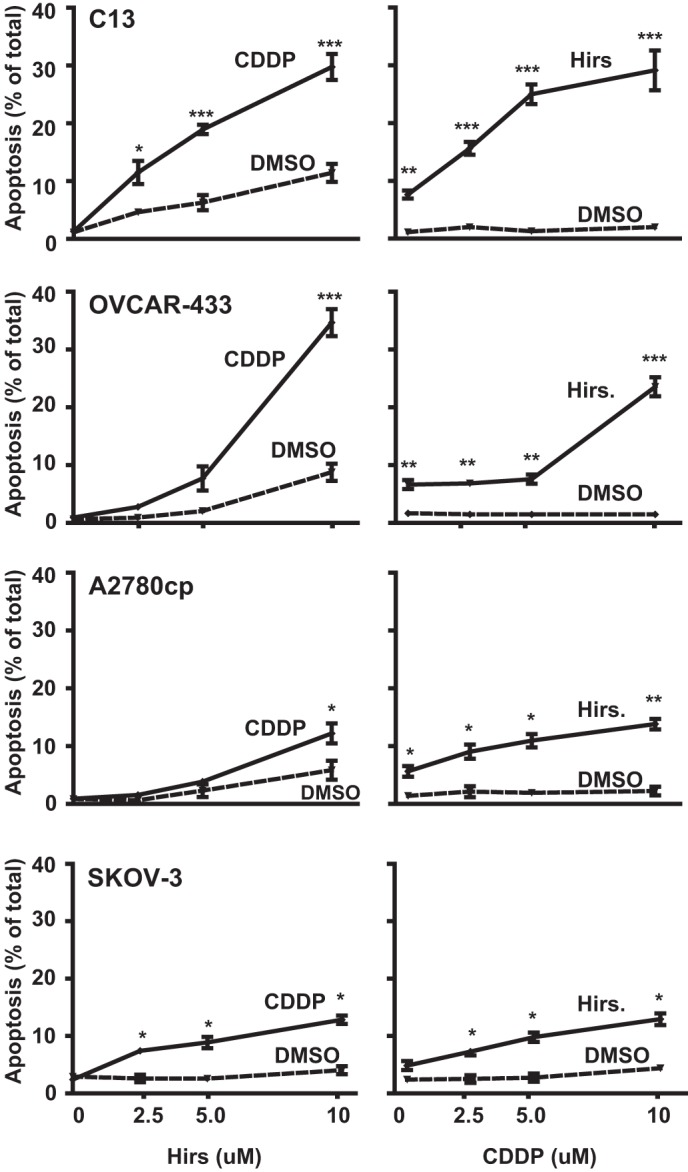
**Hirsutenone (*Hirs*) sensitizes p53 wild-type chemoresistant OVCA cells to CDDP to a greater extent than null/mutant OVCA.** *Upper four panels*, effects of CDDP (10 μm), hirsutenone (10 μm), and combined treatment on apoptosis in p53 wild-type chemoresistant OVCA. Hirsutenone treatment induces a left shift in concentration-response curves for CDDP-induced apoptosis in chemoresistant C13 and OVCAR-433. *Lower four panels*, hirsutenone-induced sensitivity in chemoresistant *p53*-mutant A2780cp* and *p53*-null SKOV3 cells at 10 μm concentration. *, *p* < 0.05; **, *p* < 0.01; ***, *p* < 0.001 *versus* respective DMSO control. *Error bars*, S.E. Apoptosis was assessed via the quantification of fragmented nuclear morphology, as described under “Experimental Procedures.”

We next investigated whether the cytotoxic activity of hirsutenone (Fig. 2) is associated with the induction of apoptosis. Wild-type *p53* (C13*, OVCAR-433) and *p53*-defective (A2780cp*, SKOV3) chemoresistant OVCA cells were cultured in the presence of hirsutenone and/or CDDP (0–10 μm; 24 h). Analysis of nuclear morphology using Hoechst 33528 revealed that hirsutenone alone caused nuclear condensation and fragmentation indicative of apoptosis in all cells except SKOV3. This response was markedly enhanced when cells were treated with CDDP and hirsutenone together (Fig. 3).

##### Reconstitution of Wild-type p53 in p53-mutant and -null OVCA Cell Lines Enhances Hirsutenone- and CDDP-induced Apoptosis

**FIGURE 4.**
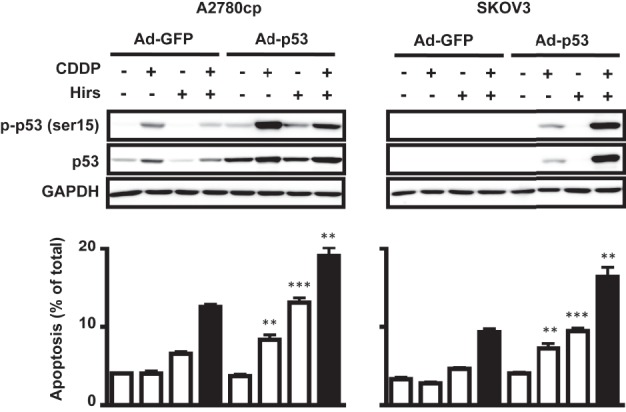
**Reconstitution of wild-type *p53* in *p53*-mutant and -null OVCA enhances hirsutenone-induced apoptosis.** *p53*-mutant (A2780cp) and *p53*-null (SKOV3) OVCA cell lines were infected with adenoviral wild-type p53 or GFP control (multiplicity of infection = 10; 24 h). The presence of wild-type p53 induced sensitivity to CDDP, while enhancing apoptosis caused by hirsutenone alone (10 μm) and in combination with CDDP (10 μm). These events occurred with concomitant activation of p53 with serine 15 phosphorylation. Apoptosis was quantified using the Hoechst assay as described under “Experimental Procedures.” **, *p* < 0.01; ***, *p* < 0.001; *error bars*, S.E.

Previous studies have underlined the central role of p53 in apoptotic processes (25). We thus investigated whether the reduced responses to hirsutenone-induced CDDP sensitivity in both *p53*-mutant (A2780cp) and *p53*-null (SKOV3) lines could be due to the lack of a functional p53. Infection of A2780cp and SKOV3 cells with adenoviral constructs containing wild-type *p53* enhanced the apoptotic response to hirsutenone and CDDP alone and in combination (Fig. 4). These findings imply that although hirsutenone can slightly but significantly enhance CDDP-induced apoptosis in a p53-independent manner, a much greater effect of the inhibitor could be realized in the presence of a functional p53.

##### Hirsutenone Modulates Phospho-Akt and Phospho-p53 Contents and Induces XIAP Proteasomal Degradation

**FIGURE 5.**
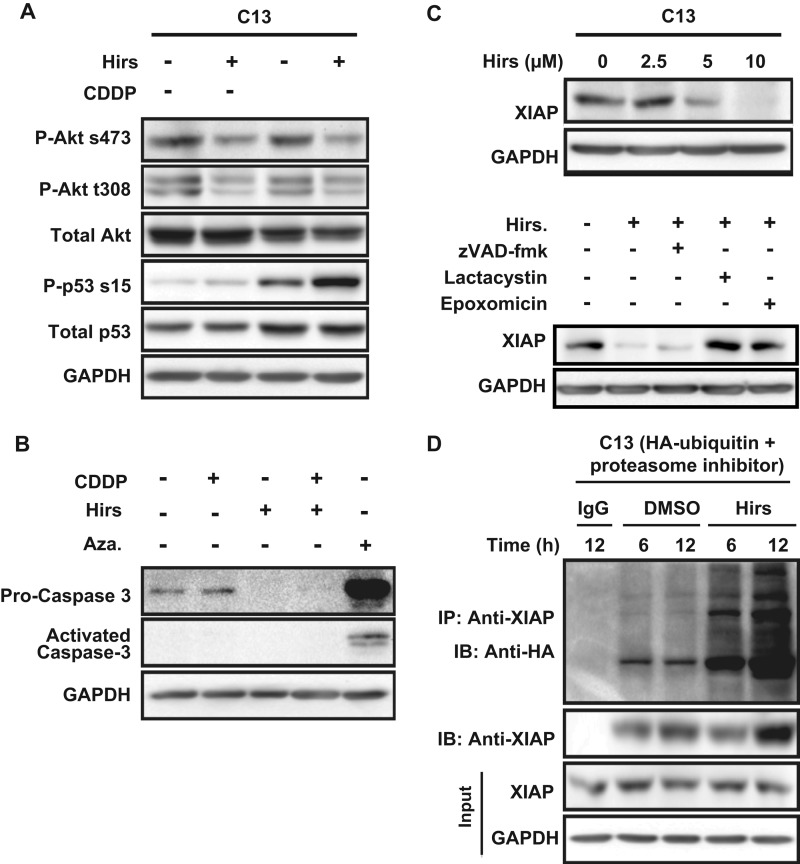
**Influence of hirsutenone (*Hirs*) on phospho-Akt, phospho-p53, caspase-3, and XIAP contents.** *A*, hirsutenone down-regulates phospho-Akt contents (Ser^473^ and Thr^308^) and increases phospho-p53 levels in chemoresistant C13 cells. Cells were treated with CDDP (10 μm) and/or hirsutenone (10 μm) for 24 h. *B*, hirsutenone affected pro- and activated caspase-3 expression in chemoresistant C13 cells. Azacytidine (*Aza*; 5 μm) was used as a positive control for activated caspase-3. *C*, hirsutenone treatment (10 μm; 24 h) down-regulates XIAP in a concentration-dependent manner in chemoresistant (C13*) OVCA. *D*, hirsutenone-induced XIAP down-regulation in chemoresistant C13 cells is attenuated by the proteasome inhibitors epoxomicin (15 nm) and lactacystin (10 μm) but not by the pan-caspase inhibitor Z-VAD-fmk (20 μm). *IP*, immunoprecipitation; *IB*, immunoblotting. *E*, immunoprecipitation hirsutenone treatment effect on XIAP ubiquitination was analyzed. C13 cells were treated as indicated after transfection with ubiquitin-HA constructs (1 μg) in the presence of the proteasome inhibitor epoxomicin (15 nm). Native XIAP protein was immunoprecipitated using a monoclonal antibody conjugated to magnetic Dynabeads (Invitrogen).

To extend the above observations, we focused our subsequent studies on p53 and Akt as two prime candidates likely to play a critical role in hirsutenone action. Whereas treatment of chemoresistant OVCA cells with CDDP (10 μm) had no detectable effect on Akt content (Fig. 5*A*), hirsutenone treatment resulted in a small but statistically significant decrease in phospho-Akt levels (Ser^473^ and Thr^308^), an effect sustained by the presence of CDDP. Similarly, CDDP alone had very little effect on p53 activation (phospho-p53 (Ser^15^)/p53 ratio), whereas this response was significantly enhanced by hirsutenone. Interestingly, combined treatment with hirsutenone and CDDP resulted in a synergistic increase in phospho-p53 content despite a decline in total p53 levels. In addition, whereas azacytidine (5 μm; 24 h), a compound known to induce cell death via reactive oxygen species generation, resulted in caspase-3 activation in C13 cells, this response was not evident in the presence of hirsutenone (Fig. 5*B*). Moreover, hirsutenone treatment decreased XIAP content in a concentration-dependent manner (Fig. 5*C*).

We next investigated whether the 26S proteasome was involved in the hirsutenone-dependent degradation of XIAP. Pretreatment of chemoresistant C13 cells with the specific proteasome inhibitors lactacystin (10 μm) or epoxomicin (15 nm) significantly attenuated hirsutenone-induced XIAP down-regulation. In contrast, the pan-caspase inhibitor Z-VAD-fmk (20 μm) was ineffective (Fig. 5*C*). An immunoprecipitation analysis revealed that hirsutenone (10 μm; 6–12 h) also induced XIAP ubiquination, suggesting that hirsutenone induces XIAP degradation via the ubiquitin-proteasome pathway (Fig. 5*D*).

##### The Effect of Hirsutenone on CDDP-induced Apoptosis in Chemoresistant OVCA Is AIF-dependent and Facilitated by XIAP-AIF Interaction

**FIGURE 6.**
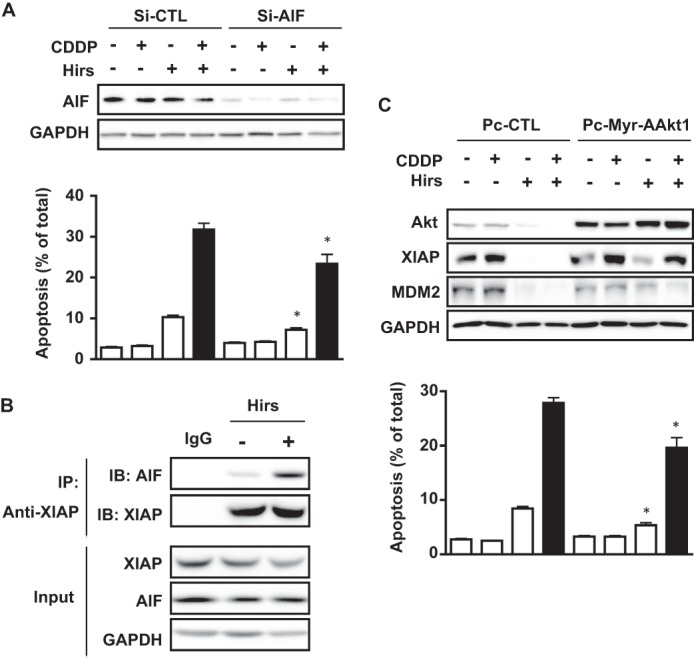
**Effects of hirsutenone (*Hirs*) on CDDP-induced apoptosis are AIF-dependent and involve XIAP-AIF interaction as well as Akt inhibition.** *A*, AIF silencing rescues C13 cells from apoptosis induced by hirsutenone and CDDP. Cells were transfected with AIF siRNA (100 nm, 48 h) or scrambled control siRNA prior to hirsutenone (10 μm) and CDDP (10 μm) treatment. Transfection with AIF siRNA significantly down-regulated AIF protein content while concomitantly attenuating apoptosis caused by hirsutenone treatment (10 μm) alone and in combination with CDDP (10 μm). *B*, immunoprecipitation (*IP*) of C13 whole cell lysate demonstrates XIAP-AIF interaction, which is enhanced by the presence of hirsutenone (10 μm). Proteins from C13 cell lysate were immunoprecipitated using anti-AIF or IgG (control antibodies) anchored to Invitrogen Dynabeads. *IB*, immunoblotting. *C*, Akt is an upstream modulator of hirsutenone action. Myristoylated AAkt1 (constitutively active Akt1) expression attenuates the cytotoxic action of CDDP (10 μm) and hirsutenone (10 μm) in C13 cells. Pc-Myr-Akt vectors were transfected into C13 cells prior to treatment as indicated. *Error bars*, S.E.

Our unexpected finding that hirsutenone failed to activate caspase-3 led us to postulate that other apoptotic mediators were involved (Fig. 5*B*). Because induction of apoptosis by AIF constitutes a major caspase-independent mechanism of apoptosis, we hypothesized that hirsutenone sensitizes chemoresistant OVCA cells to CDDP by regulating intracellular AIF level and function. To investigate whether this was the case, AIF expression was silenced with AIF-specific siRNA (100 nm; 48 h) that markedly decreased AIF protein content (Fig. 6*A*). Importantly, the down-regulation of AIF protein content markedly attenuated the levels of apoptosis induced by hirsutenone (10 μm) and hirsutenone in combination with CDDP (10 μm, 24 h).

XIAP has been reported to interact with AIF in the cytoplasm, where it degrades the protein in a ubiquitin-dependent manner (10). We further investigated whether this interaction could be playing a role in the action of hirsutenone. Immunoprecipitated XIAP revealed its physical interaction with AIF, an event that was markedly enhanced by the presence of hirsutenone (10 μm, 12 h, Fig. 6*B*). These observations lend weight to the notion that XIAP might normally be acting as an inhibitor of AIF activity, preventing its translocation to the nucleus. The ability of hirsutenone to promote XIAP degradation may therefore be one possible mechanism by which it contributes to enhanced apoptosis.

##### Hirsutenone-induced CDDP Sensitization in Chemoresistant OVCA Cells Involves Down-regulation of Akt-mediated Responses

The PI3K/Akt pathway is frequently overexpressed or hyperactivated in OVCA (26). Akt has been found to stabilize MDM2 and XIAP levels via direct phosphorylation, suppressing apoptosis induced by proapoptotic stimuli (12, 27). PI3K is a reported molecular target of hirsutenone, which may have downstream effects on Akt activity that affect modulators of chemosensitivity. To investigate this possibility, we transfected chemoresistant C13 cells with a construct expressing constitutively activated Akt1 (Pc-Myr-AAkt1). Although hirsutenone (10 μm) and CDDP (10 μm) treatment (24 h) down-regulated both XIAP and MDM2 protein content, these responses were attenuated by the expression of Myr-AAkt1, as was apoptosis induced by the two compounds (Fig. 6*C*). Taken together, these results suggest that hirsutenone enhances CDDP sensitivity, at least in part, via the inhibition of Akt and subsequent destabilization of MDM2 and XIAP.

##### Hirsutenone Promotes AIF Nuclear Translocation in the Presence of CDDP, a Response Attenuated by XIAP Overexpression

**FIGURE 7.**
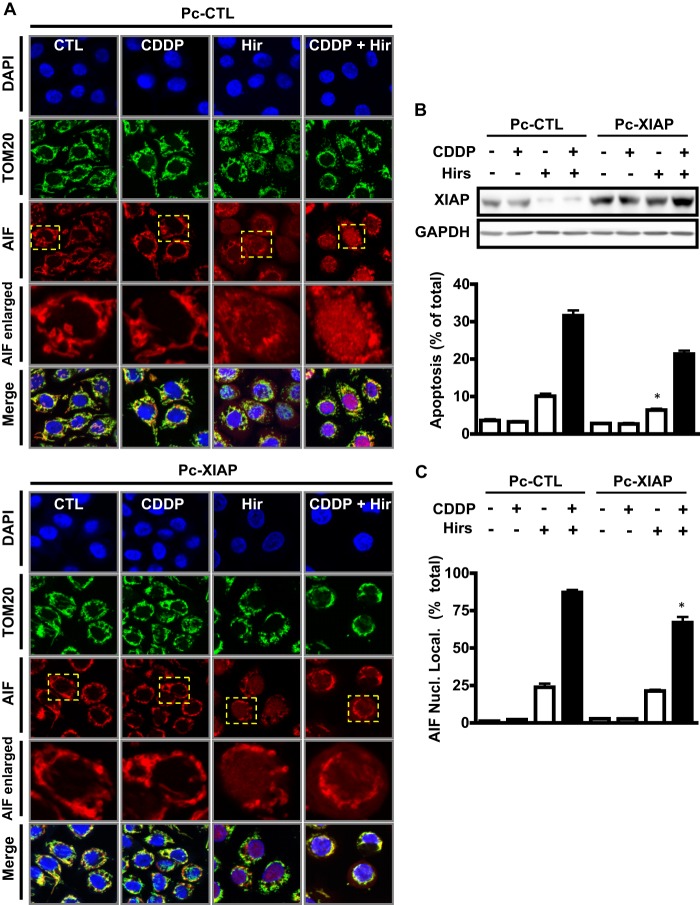
**Hirsutenone-facilitated CDDP-induced apoptosis in chemoresistant OVCA cells is mediated by AIF and by suppressed XIAP-AIF interaction.** *A*, effects of hirsutenone (*Hir*) (10 μm) and CDDP (10 μm) treatment (12 h) on AIF nuclear translocation, in the presence and absence of XIAP overexpression. C13 cells were transfected with XIAP overexpression constructs (Pc-XIAP, 0.1 μg, 48 h) or GFP control constructs (Pc-GFP, 0.1 μg, 48 h), prior to treatment. *Blue*, DAPI; *red*, AIF; *green*, TOM20 (mitochondrial membrane marker). *B*, effects of XIAP overexpression on apoptosis induced by hirsutenone (10 μm) and CDDP (10 μm) treatment (24 h). C13 cells were transfected with XIAP overexpression constructs (Pc-XIAP, 1 μg, 48 h) or GFP control constructs (Pc-GFP, 1 μg, 48 h), prior to treatment. *C*, quantification of AIF nuclear localization data shown in *A*. Nuclear signal was quantified using ImageJ software. *, *p* < 0.05; **, *p* < 0.01 *versus* respective DMSO control. *Error bars*, S.E..

We thus determined whether hirsutenone and/or CDDP could induce the translocation of AIF from the mitochondria to the nucleus. Cells were immunostained after treatment (12 h) with hirsutenone (10 μm) and/or CDDP (10 μm). In control cells, AIF clearly co-localized with the mitochondrial marker TOM20 (*green*). Upon treatment with hirsutenone, AIF is localized moderately to the nucleus (DAPI, *blue*) and less in the mitochondria (Fig. 7). This phenomenon was not evident with CDDP treatment alone, but was increased markedly in the presence of both CDDP and hirsutenone.

**FIGURE 8.**
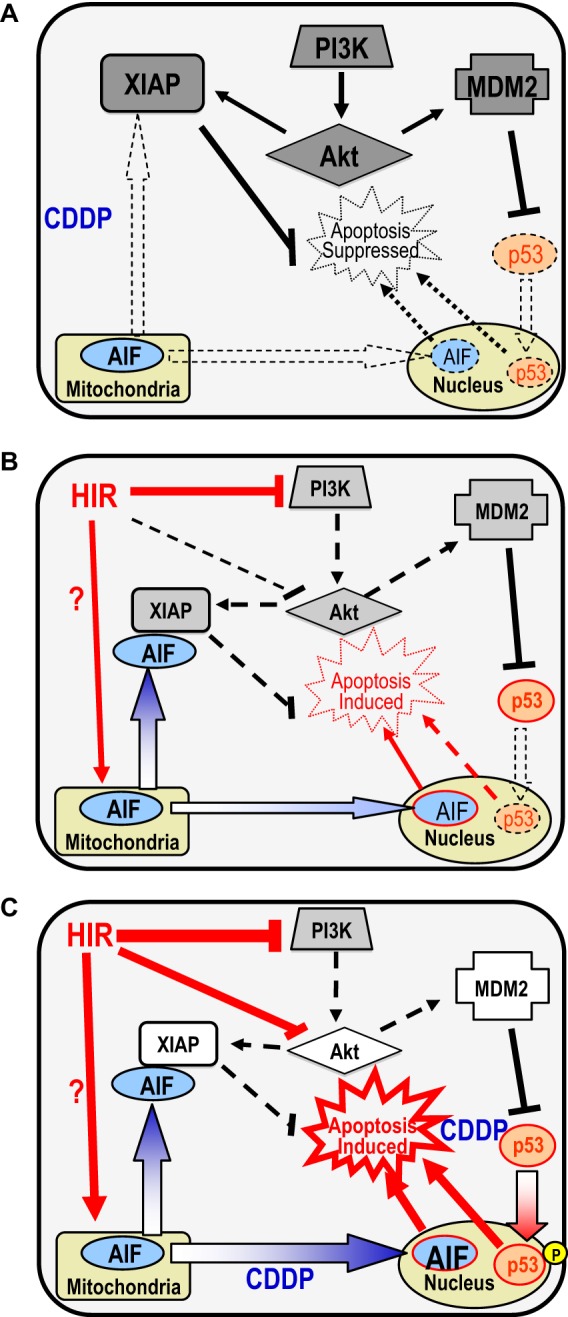
**Hypothetical model illustrating the actions of hirsutenone (*HIR*) and CDDP in chemoresistant OVCA.** Although many further aspects remain to be determined, the PI3K/Akt pathway is known to be frequently overexpressed and XIAP stabilized at high levels in chemoresistant OVCA (29). *A*, Akt appears to inhibit apoptosis by stabilizing both XIAP and MDM2 at the protein level via direct phosphorylation of these proteins (12, 30). *B*, hirsutenone suppresses Akt function by inhibiting PI3K and induces degradation of XIAP and MDM2. *C*, in the presence of CDDP, hirsutenone further enhances p53 activation, down-regulates XIAP and MDM2 content, and facilitates AIF nuclear translocation, the latter possibly through direct action of p53 at the mitochondrial level (28).

We next investigated whether the presence of XIAP would influence AIF translocation to the nucleus. Overexpression of XIAP using Pc-DNA3-XIAP vectors (1 μg; 24 h) resulted in the attenuation of AIF nuclear translocation and a concomitant decrease in apoptosis during CDDP and hirsutenone treatment (Fig. 8*A*). Taken together, these results suggest that hirsutenone-induced CDDP sensitivity in chemoresistant OVCA is dependent on AIF translocation to the nucleus, which can in turn be inhibited by XIAP.

## DISCUSSION

Chemoresistance is a major therapeutic problem and results from defects in signal transduction components that normally lead to apoptosis. Previous studies have shown that these defects primarily involve overexpressed or hyperactivated cell survival factors (including PI3K/Akt and XIAP) or down-regulated apoptosis activators (including caspases and AIF). Phytochemicals can exert influences on these factors, which could possibly lead to more desirable therapeutic outcomes. In this study, we have demonstrated that hirsutenone sensitizes chemoresistant OVCA to CDDP and described the role and regulation of AIF in hirsutenone action. Although hirsutenone could have additional cellular targets, we have demonstrated that a plausible major effector of hirsutenone action is PI3K. We hypothesize that hirsutenone directly targets PI3K *in vitro*, leading to the down-regulation of Akt activity and destabilization of MDM2 and XIAP. Akt has also been implicated to confer resistance to CDDP by modulating CDDP-induced, p53-dependent FLIP (FLICE-like inhibitory protein) ubiquitination and degradation (24), as well as facilitate the proapoptotic mitochondrial action of p53 (28).

A pressing need exists for therapeutics that can induce cell death in p53-defective cancer cells, as an estimated half of all OVCA patients carry p53 gene mutations. We observed that p53 wild-type chemoresistant cells (C13, OVCAR-433) are generally more responsive to hirsutenone treatment than those lacking a functional p53 (A2780cp, Hey, Occ-1, SKOV3). Although the reconstitution of wild-type p53 improved hirsutenone action in both A2780cp and SKOV3, the fact that hirsutenone was able to induce sensitivity in the first place, albeit by a modest amount, is evidence of the existence of p53-independent effects. Hirsutenone may therefore have clinical potential for the treatment of OVCA irrespective of p53 status.

We have determined that hirsutenone enhances p53 up-regulation and phosphorylation at Ser^15^ in the presence of CDDP. Previous work by our group has shown that this could have implications for apoptosis via the proapoptotic mitochondrial action of p53 (28), which may facilitate a more potent release of AIF for subsequent translocation to the nucleus. In addition, our results show that XIAP interacts with AIF, a response increased by the presence of hirsutenone (Fig. 6*B*). These findings raise the possibility that hirsutenone could facilitates AIF-dependent apoptosis by inducing proteasomal XIAP degradation. However, in nonstressed conditions (*i.e.* not during apoptotic induction) AIF is localized to the mitochondria where it has minimal interaction with cytoplasmic XIAP. During apoptotic induction by hirsutenone AIF is then released into the cytoplasm, where it interacts with XIAP and traverses to the nucleus and induces apoptosis. This is partially supported by the localization data in Fig. 7. During such release, AIF in the cytoplasm is prone to XIAP interaction and degradation (if XIAP is present); however, concomitant release of other mitochondrial proteins such as ARTS (apoptosis-related protein in the TGF-β signaling pathway) might be the cause of the down-regulation of XIAP protein content (31). This could explain the increase in XIAP-AIF interaction during hirsutenone challenge, which is due to AIF release from the mitochondria, but occurs with concomitant release of ARTS, thereby causing overall levels of XIAP to decline.

Interestingly, it appeared that hirsutenone treatment, rather than activate caspase-3, to some extent appeared to down-regulate protein content of the procaspase form. Although the mechanisms responsible for such an outcome remain to be determined, the possibility exists that hirsutenone may act on other intracellular regulators which could lead to the degradation of procaspase-3 or decrease the transcription of its mRNA. It also remains to be determined whether hirsutenone perturbs cell cycle progression, and we acknowledge that cell cycle redistribution potentially caused by treatment may induce changes in Akt activity. Our study primarily focused on the influence of apoptotic regulators (p53, caspase-3, AIF, XIAP).

Although a number of the other compounds we tested (piceatannol, cyanidin, and delphinidin) were also potent inhibitors of PI3K activity, their effects *in vitro* were less than expected. This may suggest that hirsutenone has additional molecular targets, in addition to PI3K. It is also possible that although such compounds can inhibit PI3K activity in a cell-free system, their effects on cell line behavior are less distinct due to physical attributes or possible involvement of other determinants of chemoresistance, including XIAP and FLIP (11, 24). We also noted that hirsutenone was less effective in chemosensitive cells. This is not surprising because chemosensitive cells are generally less reliant on the PI3K/Akt pathway than their chemoresistant counterparts.

Although we have determined some factors involved, many aspects of the hirsutenone mode of action during OVCA treatment remain unclear (Fig. 8). It would therefore be possible that hirsutenone treatment could, act to release AIF from the mitochondria via its activation of p53, or an unidentified mechanism. Whether this is indeed the case requires further investigation.

In summary, we have determined that hirsutenone enhances CDDP-dependent apoptosis in chemoresistant OVCA by down-regulating XIAP through degradation via the proteasome-ubiquitin pathway and by inducing AIF translocation to the nucleus. The greater induction of apoptosis associated with hirsutenone treatment was found to be, at least in part, due to the ability of hirsutenone to inhibit PI3K/Akt function. A better understanding of the pharmacodynamic properties and *in vivo* stability of hirsutenone is needed. Further *in vivo* studies will provide better insights into the possible application of hirsutenone treatment for chemoresistant ovarian cancer.

## Footnotes

2 The abbreviations used are:

OVCA: ovarian cancer
AIF: apoptosis-inducing factor
CDDP: cisplatin
DMSO: dimethyl sulfoxide
fmk: fluoromethyl ketone
TEMED: *N*,*N*,*N*′,*N*′-tetramethylethylenediamine
XIAP: X-linked inhibitor of apoptosis
Z: benzyloxycarbonyl.
